# Geniposide Attenuates Hyperglycemia-Induced Oxidative Stress and Inflammation by Activating the Nrf2 Signaling Pathway in Experimental Diabetic Retinopathy

**DOI:** 10.1155/2021/9247947

**Published:** 2021-12-13

**Authors:** Yuanyuan Tu, Lele Li, Linling Zhu, Yang Guo, Shu Du, Yuxing Zhang, Zhenzhen Wang, Yuting Zhang, Manhui Zhu

**Affiliations:** ^1^Department of Ophthalmology, Lixiang Eye Hospital of Soochow University, Suzhou, Jiangsu, China; ^2^Department of Ophthalmology, Affiliated Hospital 2 of Nantong University, Nantong, Jiangsu, China; ^3^Department of Internal Medicine, Xiangcheng Distirict Hospital of Chinese Medicine, Suzhou, Jiangsu, China

## Abstract

Geniposide (GEN) is a natural antioxidant and anti-inflammatory product and plays an important role in the treatment of diabetes and diabetic complications. To explore the biological functions and mechanism of GEN in diabetic retinopathy (DR), we constructed the in vitro and in vivo model of DR by using primary cultured mouse retinal Müller cells and C57BL/6 mice, respectively. We found that GEN inhibited ROS accumulation, NF-*κ*B activation, Müller cell activation, and inflammatory cytokine secretion both in vitro and in vivo, which is probably mediated through the Nrf2 pathway. Exendin (9-39) (EX-9), an antagonist of glucagon-like peptide-1 receptor (GLP-1R), abolished the protective effect of GEN on high glucose- (HG-) induced Müller cells. Additionally, GEN decreased hyperglycemia-induced damage to Müller cells and blood-retinal barrier in the retinas of mice with DR. We demonstrated that GEN was capable of protecting Müller cells and mice from HG-induced oxidative stress and inflammation, which is mostly dependent on the Nrf2 signaling pathway through GLP-1R. GEN may be an effective approach for the treatment of DR.

## 1. Introduction

Diabetes is one of the most important and serious global health problems worldwide. Epidemiological studies have shown that the prevalence of diabetes will increase to 7.7% and affect 439 million adults by 2030 [1]. Diabetes causes serious damage to microvessels and macrovessels and leads to vascular complications in the human body [2]. Among microvascular complications, diabetic retinopathy (DR) remains one of the most serious and common diabetes-associated complications. As the main glial cells in the retina, Müller cells play a crucial role in the progression of DR. Our previous studies showed that Müller cells become activated and secrete several inflammatory cytokines in experimental DR model. Inhibition of Müller cell gliosis may decrease damage to the blood-retinal barrier (BRB) and reduce the loss of retinal ganglion cells (RGCs) [3, 4]. Thus, preventing Müller cell gliosis and subsequent inflammatory factor production may be an effective therapeutic strategy for DR treatment.

Chronic hyperglycemia-induced oxidative stress and low-grade inflammation are thought to play crucial roles in the onset and development of DR [5]. When there is an imbalance between excessive reactive oxygen species (ROS) production and the ability of endogenous antioxidant factors to clear ROS, oxidative stress occurs [6]. Oxidative stress is characterized by ROS-induced overexpression of proinflammatory and proangiogenic factors, which damage glial cells, vascular cells, and neurons [7, 8]. Cumulative evidence has indicated that ROS plays an important role in activating transcription factor nuclear factor-kappa B (NF-*κ*B) [9, 10]. NF-*κ*B is triggered and translocated to the nucleus where it activates the transcription of proinflammatory cytokines, such as tumor necrosis factor-*α* (TNF-*α*), interleukin-6 (IL-6), and interleukin-1*β* (IL-1*β*) [11, 12].

The interaction of retinal glial cells and blood vessels is important for maintaining the homeostasis and survival of retinal tissue [13]. Müller cells are the main glial cells in the retina, and under hyperglycemic conditions, they undergo oxidative damage and exhibit a reactive phenotype, which is manifested by the upregulation of glial fibrillary acidic protein (GFAP) expression and the subsequent production of proinflammatory factors [14, 15]. In addition, Müller cells span the entire thickness of the retina, and the anatomical association of Müller cells with neurons and microvessels means that damage to Müller cells will lead to severe injury to neurons and blood vessels [16–18]. Therefore, inhibiting oxidative stress and inflammation in Müller cells in a hyperglycemic environment may improve retinal vascular and nerve damage to alleviate DR progression.

Geniposide (GEN) is a natural product extracted from gardenia fruit that has a variety of biological properties, such as antioxidant and anti-inflammatory activities [19, 20]. Studies have revealed that GEN protects against myocardial ischemia reperfusion injury in diabetic rats by suppressing oxidative stress through the nuclear factor erythroid 2-related factor (Nrf2)/heme oxygenase-1 (HO-1) signaling pathway [3]. In ApoE^−/−^ mice and RAW264.7 cells, GEN treatment decreased the areas of atherosclerotic plaques and the production of inflammatory cytokines, and the anti-inflammatory mechanism was related to the miR-101/mitogen-activated protein kinase phosphatase-1/P38 signaling pathway [21]. The metabolite of GEN, genipin, leads to HO-1 upregulation and participates in the anti-inflammatory response, which is mediated by PI3 kinase and activation of the downstream targets JNK1/2 and Nrf2 [22]. Studies have suggested that GEN plays a vital antioxidant and anti-inflammatory role and is closely associated with the Nrf2 signaling pathway. Moreover, as a novel agonist of glucagon-like peptide-1 receptor (GLP-1R), GEN also has a role in the treatment of diabetes and diabetic complications [23–25]. However, the effect of GEN on DR pathogenesis and whether Nrf2 is involved remain unknown. Therefore, the purpose of our study was to explore the role of GEN in DR development and the underlying mechanisms.

## 2. Materials and Methods

### 2.1. Cell Culture

Mouse primary retinal Müller cells were isolated from 3-day-old newborn C57BL/6 pups. The mice were obtained from the Experimental Animal Center of Soochow University. Müller cells were extracted and identified as previously described [26]. Retinal Müller cells were cultured in DMEM supplemented with 10% fetal bovine serum (FBS, Gibco), streptomycin (100 mg/ml), and penicillin (100 U/ml) (Gibco). Then, the cells were cultured in a humidification incubator (5% CO_2_) at 37°C, and the medium was replaced every two days.

### 2.2. Cell Treatments

The cells were treated with 5 mM D-glucose (normal glucose, NG) or 30 mM D-glucose (HG) (#310808; Sigma, USA) for 24 h to mimic the diabetic environment before or after certain experiments. Different concentrations of GEN (#SML0153; Sigma, USA) (25, 50, 100, and 200 *μ*g/ml) were used to treat Müller cells for 24 h before HG was added. The dose used of GEN was based on a previous study [27]. The ROS scavenger N-acetylcysteine (NAC) (1 mM, 24 h) (#A7250, Merck, USA) [26] and GLP-1R antagonist exendin (9-39) (EX-9) (200 nmol/l, 1 h) (#ab141101, Abcam, UK) [28] were used to treat the cells before HG or GEN administration. After certain treatments, the cell culture medium was collected and stored at -80°C for cytokine analysis.

### 2.3. Cell Viability Assay

Müller cells were seeded in 96-well plates at a density of 5 × 10^3^ cells/well. After 24 h, the cells were treated with different concentrations of GEN (25, 50, 100, and 200 *μ*g/ml) with or without HG for 24 h. Then, cell viability was measured by a Cell Counting Kit-8 (CCK-8, #CK04, Dojindo, Japan) according to the manufacturer's instructions. Briefly, after treatment, Müller cells were washed with Hank's solution, and 100 *μ*l of medium supplemented with 10 *μ*l of CCK-8 solution was added to each well. After being incubated for 2 h in a CO_2_ incubator, the absorbance at 450 nm was measured by a Thermo MultiSkan GO microplate reader (Thermo Fisher, USA).

### 2.4. Immunofluorescence Analysis

Müller cells were seeded in the slide chamber at a density of 1 × 10^3^ cells per well. After the indicated treatments, the cells were fixed with 4% paraformaldehyde (PFA) at room temperature for 30 min. The retinal patch was first isolated from the mouse retina and fixed at room temperature with PFA for 1 h. After being blocked and permeabilized, the slides were incubated with nuclear factor kappa B (NF-*κ*B) p65 antibodies (host species: rabbit; species reactivity: mouse, human; dilution: 1 : 1000; #ab16502, Abcam), and the retinas were incubated with isolectin B4 (1 : 1000, #I21411, Invitrogen) and GFAP (host species: rabbit; species reactivity: mouse, rat; dilution: 1 : 1000, #ab7260, Abcam) antibodies at 4°C overnight. Then, the samples that had been incubated with NF-*κ*B p65 and GFAP antibodies were incubated with secondary antibodies (1 : 200 dilution) in the dark at room temperature for 2 h. Finally, the cells were stained with DAPI at room temperature for 15 min and observed by confocal laser fluorescence microscopy (SP8, Leica).

### 2.5. Animals

Male C57BL/6 mice (8-weeks old) were purchased from the Laboratory Animal Center of Soochow University. The mice were housed in standard pathogen-free conditions and were randomly divided into 6 groups: normal, normal+PBS, normal+GEN, DM, DM+PBS, and DM+GEN. The diabetic mouse model was established as previously described [3]. Briefly, the mice were fasted for 12 hours before streptozotocin (STZ) injection. Then, the mice received intraperitoneal injections of 50 mg of STZ once per day for 5 consecutive days. Mice with a blood glucose greater than 16.7 mmol/l were regarded as diabetic and were used in follow-up experiments. The mice in normal group were given an intraperitoneal injection of the same amount of citrate buffer. Four weeks after successful modeling, the mice in the GEN treatment group were given tail vein injections of GEN (50 mg/kg/day) for one week. GEN was dissolved in phosphate-buffered saline (PBS), and equal volumes of PBS without GEN were injected as the solvent control. Finally, the mice were sacrificed for certain experiments. All animal experiments were approved by the Animal Research Ethics Committee of Soochow University and were in accordance with the Chinese National Standard.

### 2.6. Intracellular ROS Detection

The generation of ROS was measured by an ROS assay kit (#S0033M, Beyotime, China). In vitro, Müller cells were seeded on 24-well plates and incubated with 2,7-dichlorodi-hydrofluorescein diacetate (DCFH-DA), at a concentration of 10 *μ*M for 20 min at 37°C. The cells were washed three times in serum-free medium to completely remove unincorporated DCFH-DA. The level of ROS in the retinas of mice was measured as previously described [29]. ROS levels were measured by a immunofluorescence microscope (Leica).

### 2.7. Western Blotting

Total protein was extracted from cultured Müller cells and mouse retinas with protein lysis buffer. Cytoplasmic and nuclear proteins were extracted from cells using a PARIS Kit (#AM1556, Life Technologies, USA) according to the manufacturer's instructions. The protein concentration was measured by a BCA assay kit (#P0012S, Beyotime, China). Equal amounts of proteins in each sample were separated by SDS-PAGE and then transferred to PVDF membranes. After being blocked, the membranes were hybridized with primary antibodies against I*κ*B*α* (1 : 1000 dilution, #ab7217, Abcam), p-I*κ*B*α* (1 : 1000 dilution, #2859, Cell Signaling Technology, USA), NF-*κ*B P65 (1 : 2000 dilution, #ab16502, Abcam), p-P65 (1 : 1000 dilution, #3031, Cell Signaling Technology), GFAP (1 : 3000 dilution, #ab7260, Abcam), Nrf2 (1 : 1000 dilution, #ab92946, Abcam), *β*-actin (1 : 1000 dilution, #ab8226, Abcam), histone H3 (H3) (1 : 1000 dilution, #ab6147, Abcam), HO-1 (1 : 2000 dilution, #ab189491, Abcam), NAD(P)H quinone dehydrogenase 1 (NQO1) (1 : 1000 dilution, #ab34173, Abcam), and GAPDH (1 : 5000 dilution, #ab8245, Abcam) at 4°C overnight. *β*-Actin and H3 were used as cytoplasmic and nuclear internal controls, respectively. The membranes were then washed and hybridized with horseradish peroxidase-conjugated secondary antibodies (Cell Signaling Technology) at room temperature for 1 h. The protein bands were detected with a chemiluminescence reagent and visualized with a Bio-Rad imaging system (Bio-Rad Laboratories, Hercules, CA, USA). ImageJ software was used to quantify the band intensities, and GAPDH was used as the loading control.

### 2.8. Quantitative Real-Time PCR (qRT-PCR)

Total RNA was extracted with TRIzol reagent (#15596018, Invitrogen, USA) according to the manufacturer's instructions. Total RNA (1 *μ*g) was synthesized into cDNA using a Revert Aid First Strand cDNA Synthesis Kit (#K1622, Thermo Scientific, USA). The sequences of the qRT-PCR primers were as follows: VE-cadherin: forward primer 5′-TGGAAGGTCTGCACCTGCTA-3′, reverse primer 5′-TTTGGCCCACGGGATTG-3′; claudin-5: forward primer 5′-TCTGCTGGTTCGCCAACAT-3′, reverse primer 5′-CGGCACCGTCGGATCA-3′; occludin: forward primer 5′-TGTGGGATAAGGAACACATTTATGA-3′, reverse primer 5′-CAGACACATTTTTAACCCACTCTTCA-3′; and ZO-1: forward primer 5′-TGAACGCTCTCATAAGCTTCGTAA-3′, reverse primer 5′-ACCGTACCAACCATCATTCATTG-3′. PowerUP SYBR Green Master Mix (#A25742, Thermo Scientific) was used to detect the transcription products of the cDNA samples on an ABI 7500 Real-Time PCR system (Foster City, USA). GAPDH was used as the internal control, and the 2^−ΔΔCT^ method was used to calculate the relative expression of the target genes.

### 2.9. Enzyme-Linked Immunosorbent Assay (ELISA)

The concentrations of TNF-*α* (#BMS607-3, Invitrogen), IL-1*β* (#PI301, Beyotime, China), and IL-6 (#BMS603-2, Invitrogen) in the cell culture supernatant and retinas were measured by commercial ELISA kits according to the manufacturers' instructions. By measuring the optical density of each well at 450 nm, the concentrations of these cytokines were quantified with reference to the standard curve.

### 2.10. Cell Transfection

Nrf2 siRNA and scramble siRNA were purchased from RIBO Biology Company (Nrf2 siRNA, 5′-UGAAAGCACAGCAGAAUUTT-3′). According to the manufacturer's instructions, Lipofectamine 2000 transfection reagent (#11668019, Invitrogen, USA) was used to perform the cell transfections.

### 2.11. Statistical Analysis

All data are presented as the mean ± SEM. GraphPad Prism version 7 software (GraphPad, USA) was used for statistical analysis. All the experiments in our study were repeated at least three times. Student's *t*-test (2-group comparisons) and one-way ANOVA followed by Tukey's multiple comparison posttest (multiple-group comparisons) were used to assess whether there was a significant difference between the groups.

## 3. Results

### 3.1. ROS Accumulation Promotes NF-*κ*B Activation, Followed by Glial Activation and Inflammatory Cytokine Secretion in HG-Stimulated Müller Cells

**Figure 1:**
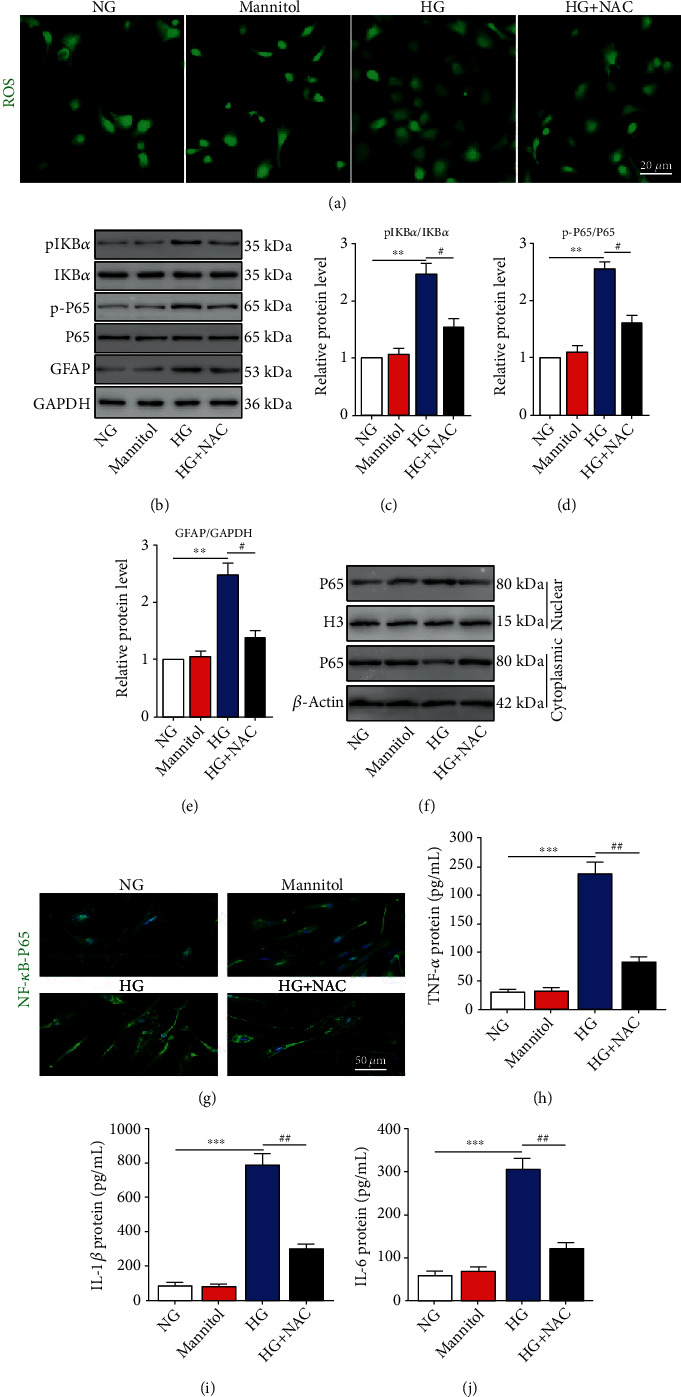
Reactive oxygen species (ROS) accumulation promotes NF-*κ*B activation, followed by glial reactivation and inflammatory cytokine secretion in HG-stimulated Müller cells. Müller cells were stimulated with normal glucose (NG) and high glucose (HG) to establish an in vitro diabetes model, and mannitol was used as the osmotic control. Cells were pretreated with N-acetylcysteine (NAC) before HG administration. (a) ROS accumulation was measured by an ROS assay kit. (b–e) The protein expression of I*κ*B*α*, p-I*κ*B*α*, P65, p-P65, and glial fibrillary acidic protein (GFAP) was assayed by western blotting. ^∗∗^*P* < 0.01 vs. the NG group and ^#^*P* < 0.05 vs. the HG group. (f) The protein expression of P65 in the nucleus and cytoplasm was measured by western blotting. (g) Immunofluorescent staining of NF-*κ*B P65 in Müller cells. (h–j) ELISA was performed to measure the protein levels of TNF-*α*, IL-1*β*, and IL-6 in Müller cells. ^∗∗∗^*P* < 0.001 vs. the NG group and ^##^*P* < 0.01 vs. the HG group. *n* = 4/group.

As shown in Figure 1(a), intracellular ROS levels were increased under HG conditions and decreased by the ROS scavenger NAC (Figure 1(a)). After HG stimulation, the phosphorylation of the NF-*κ*B-related proteins I*κ*B*α* and P65 was significantly upregulated, and this effect was reversed by NAC pretreatment (Figures 1(b)–1(d)). We also used western blot and immunofluorescent staining to determine the subcellular localization of NF-*κ*B P65 in Müller cells. We found that NF-*κ*B P65-positive staining was mainly localized in the cytoplasm in the control group and was translocated to the nucleus by HG stimulation. Interestingly, the HG-induced translocation of NF-*κ*B P65 was inhibited in the presence of NAC (Figure 1(g)). Additionally, HG promoted the expression of the glial activation marker GFAP (Figures 1(b) and 1(e)) and inflammatory factors, including TNF-*α*, IL-1*β*, and IL-6 (Figures 1(h)–1(j)), while NAC treatment counteracted the effect of HG. These data suggest that HG promotes ROS accumulation and induces the NF-*κ*B signaling pathway activation, thereby increasing glial activation and inflammatory cytokine secretion by HG-stimulated Müller cells.

### 3.2. GEN Is Not Cytotoxic to Müller Cells within a Range of Concentrations

**Figure 2:**
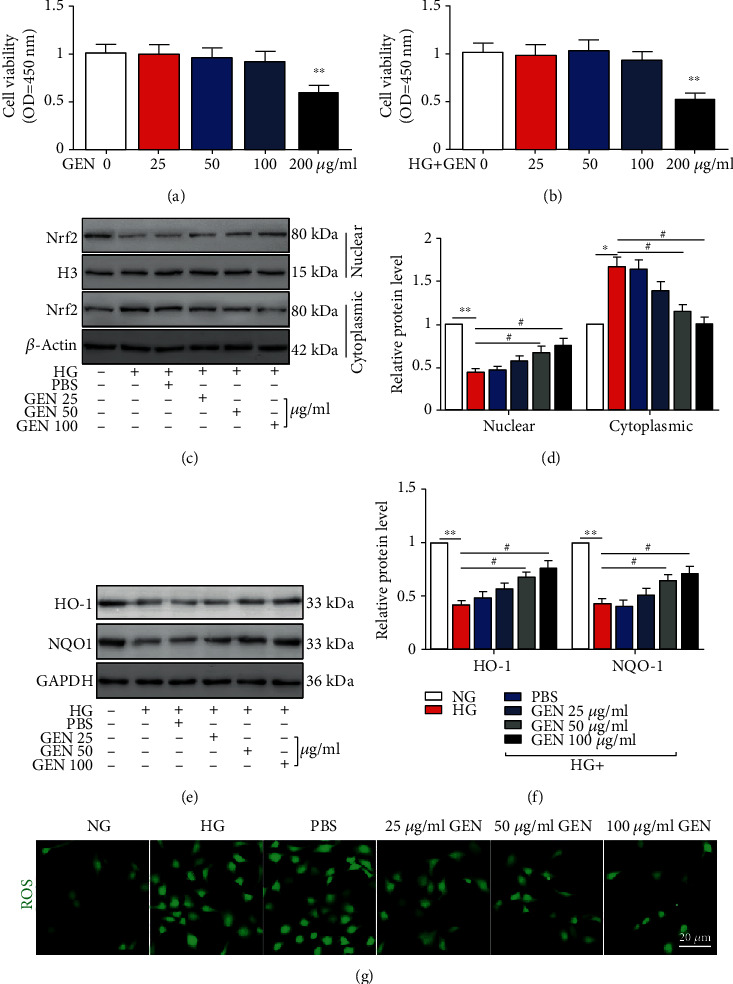
The effects of geniposide (GEN) on viability, Nrf2 nuclear translocation, and ROS accumulation in HG-stimulated Müller cells. Müller cells were exposed to different concentrations of GEN with or without HG. (a, b) The viability of Müller cells was measured by CCK-8 assays. ^∗∗^*P* < 0.01 vs. the control group. (c, d). The protein expression of Nrf2 in the nucleus and cytoplasm was measured by western blotting. H3 and *β*-actin were used as nuclear and cytoplasmic loading controls, respectively. ^∗^*P* < 0.05 and ^∗∗^*P* < 0.01 vs. the NG group and ^#^*P* < 0.05 vs. the HG group. (e, f) The protein expression of HO-1 and NQO1 was measured by western blotting. ^∗∗^*P* < 0.01 vs. the NG group and ^#^*P* < 0.05 vs. the HG group. (g) ROS production was measured by an ROS assay kit. *n* = 4/group.

We next examined the cytotoxicity of GEN on Müller cells under normal or HG conditions. As shown in Figures 2(a) and 2(b), 25, 50, and 100 *μ*g/ml GEN did not inhibit Müller cell viability, while 200 *μ*g/ml GEN suppressed cell viability under both normal and HG conditions (Figures 2(a) and 2(b)).

### 3.3. GEN Induces Nrf2 Nuclear Translocation and Inhibits ROS Accumulation in HG-Stimulated Müller Cells

We also found that GEN treatment dose-dependently induced Nrf2 nuclear translocation (Figures 2(c) and 2(d)). Moreover, the Nrf2-targeted antioxidant genes HO-1 and NQO1 were also assessed. As shown in Figures 2(e) and 2(f), the expression of HO-1 and NQO1 in Müller cells was markedly decreased under HG conditions and dose-dependently increased by GEN treatment. HG stimulation exacerbated ROS accumulation. Conversely, GEN significantly reduced HG-induced ROS levels (Figure 2(g)).

### 3.4. GEN Inhibits NF-*κ*B Activation and HG-Induced Glial Activation and Inflammatory Cytokine Secretion in Müller Cells

**Figure 3:**
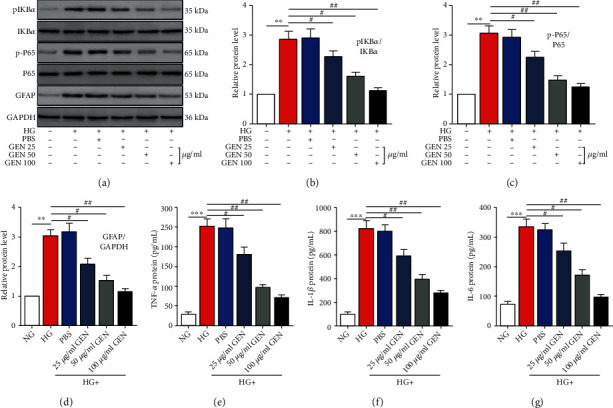
GEN inhibits NF-*κ*B activation and HG-induced glial activation and inflammatory cytokine secretion in Müller cells. (a–d) The protein expression of I*κ*B*α*, p-I*κ*B*α*, P65, p-P65, and GFAP was measured by western blotting. ^∗∗^*P* < 0.01 vs. the NG group and ^#^*P* < 0.05 and ^##^*P* < 0.01 vs. the HG group. (e–g) ELISA was used to measure the protein levels of TNF-*α*, IL-1*β*, and IL-6 in Müller cells. ^∗∗∗^*P* < 0.001 vs. the NG group and ^#^*P* < 0.05 and ^##^*P* < 0.01 vs. the HG group. *n* = 4/group.

The inhibitory effect of GEN on ROS accumulation prompted us to investigate whether GEN was involved in HG-induced NF-*κ*B activation, glial activation, and inflammatory cytokine secretion. We found that the phosphorylated forms of the NF-*κ*B-related proteins I*κ*B*α* and P65 were significantly upregulated after HG stimulation, and this effect was dose-dependently reversed by GEN pretreatment (Figures 3(a)–3(c)). Additionally, HG promoted the expression of GFAP and the secretion of inflammatory cytokines, including TNF-*α*, IL-1*β*, and IL-6, while GEN treatment reversed HG-mediated promotion of gliosis (Figures 3(a) and 3(d)) and inflammatory cytokine secretion (Figures 3(e)–3(g)).

### 3.5. GEN Inhibits NF-*κ*B Activation, Müller Cell Activation, and Inflammatory Cytokine Secretion through the Nrf2 Antioxidant Pathway

**Figure 4:**
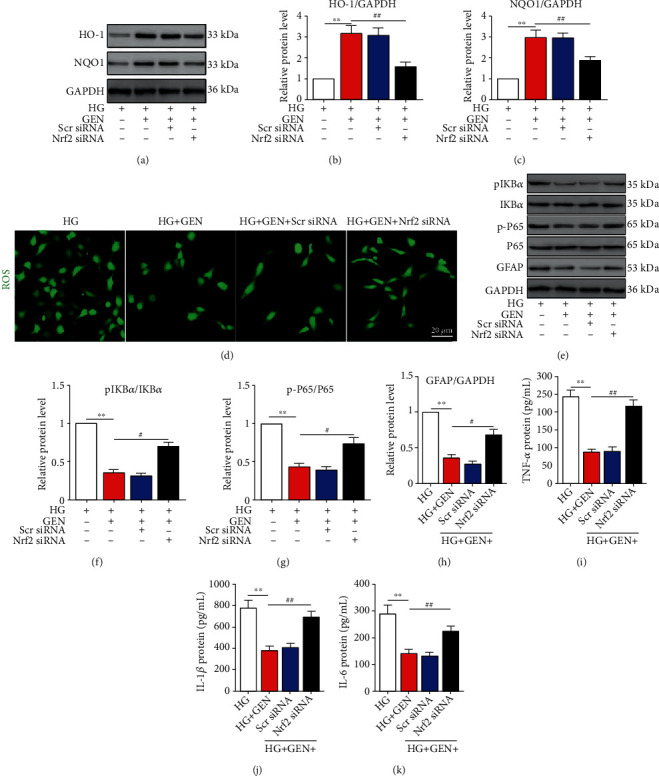
GEN inhibits NF-*κ*B activation, Müller cell activation, and inflammatory secretion through the Nrf2 antioxidant pathway. Nrf2 siRNA transfection was used to knockdown Nrf2 expression, and scramble siRNA (Scr siRNA) was used as the negative control. GEN was administered to Müller cells 24 h prior to stimulation with HG. (a–c) The protein expression of HO-1 and NQO1 was measured by western blotting. ^∗∗^*P* < 0.01 vs. the HG group and ^#^*P* < 0.05 vs. the HG+GEN group. (d) ROS production was measured by an ROS assay kit. (e–h) The protein expression of I*κ*B*α*, p-I*κ*B*α*, P65, p-P65, and GFAP was measured by western blotting. ^∗∗^*P* < 0.01 vs. the HG group and ^#^*P* < 0.05 vs. the HG+GEN group. (i–k) ELISA was used to measure the protein levels of TNF-*α*, IL-1*β*, and IL-6 in Müller cells. ^∗∗^*P* < 0.01 vs. the HG group and ^##^*P* < 0.01 vs. the HG+GEN group. *n* = 4/group.

To confirm whether the anti-inflammatory effect of GEN was mediated by activating the Nrf2 antioxidant pathway, we investigated the effect of Nrf2 knockdown on oxidative stress and inflammation in HG-stimulated Müller cells. GEN was used at a concentration of 100 *μ*g/ml in the subsequent experiments due to its improved protective effects. The results suggested that GEN treatment significantly promoted the expression of HO-1 and NQO1, and this effect was reversed by downregulating Nrf2 (Figures 4(a)–4(c)). Conversely, GEN decreased ROS accumulation in HG-stimulated Müller cells, and Nrf2 knockdown blocked the inhibitory effect of GEN on ROS accumulation (Figure 4(d)). In addition to cellular oxidative stress, Nrf2 knockdown also suppressed the effect of GEN on the NF-*κ*B pathway activation (Figures 4(e)–4(g)), expression of the glial activation marker GFAP (Figures 4(e) and 4(h)), and inflammation-associated cytokine secretion (Figures 4(i)–4(k)).

### 3.6. The Protective Effects of GEN on HG-Stimulated Müller Cells Were Abolished by a GLP-1R Antagonist

**Figure 5:**
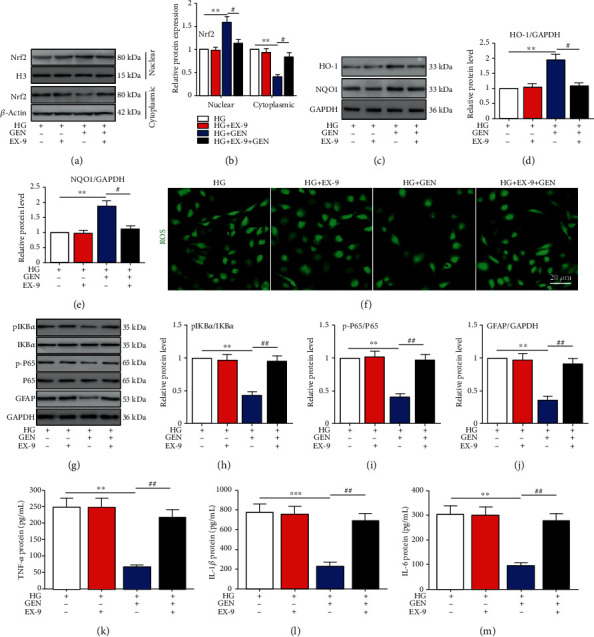
The protective effects of GEN on HG-stimulated Müller cell activation were abolished by a GLP-1R antagonist. Müller cells were pretreated with exendin (9-39) (EX-9) before HG or GEN administration. (a, b) The protein expression of Nrf2 in the nucleus and cytoplasm was measured by western blotting. ^∗∗^*P* < 0.01 vs. the HG group and ^#^*P* < 0.05 vs. the HG+GEN group. (c–e) The protein expression of HO-1 and NQO1 was measured by western blotting. ^∗∗^*P* < 0.01 vs. the HG group and ^#^*P* < 0.05 vs. the HG+GEN group. (f) ROS production was measured by an ROS assay kit. (g–j) The protein expression of I*κ*B*α*, p-I*κ*B*α*, P65, p-P65, and GFAP was measured by western blotting. ^∗∗^*P* < 0.01 vs. the HG group and ^##^*P* < 0.01 vs. the HG+GEN group. (k–m) ELISA was used to measure the protein levels of TNF-*α*, IL-1*β*, and IL-6 in Müller cells. ^∗∗^*P* < 0.01 and ^∗∗∗^*P* < 0.001 vs. the HG group and ^##^*P* < 0.01 vs. the HG+GEN group. *n* = 4/group.

To further investigate the mechanism of GEN, we evaluated the effects of the GLP-1R antagonist EX-9 on oxidative stress and inflammation in Müller cells. GEN increased the nuclear translocation of Nrf2, and EX-9 inhibited the effect of GEN (Figures 5(a) and 5(b)). Similarly, EX-9 eliminated the GEN-induced increases in HO-1 and NQO1 (Figures 5(c)–5(e)). In addition, GEN suppressed ROS accumulation (Figure 5(f)), NF-*κ*B pathway activation (Figures 5(g)–5(i)), expression of the glial activation marker GFAP (Figure 5(j)), and inflammation-associated cytokine secretion (Figures 5(k)–5(m)), and these effects were reversed by EX-9.

### 3.7. GEN-Mediated Activation of the Nrf2 Signaling Pathway Reduces NF-*κ*B Activation and Decreases GFAP Production and Inflammatory Cytokine Secretion in the Retinas of Diabetic Mice

**Figure 6:**
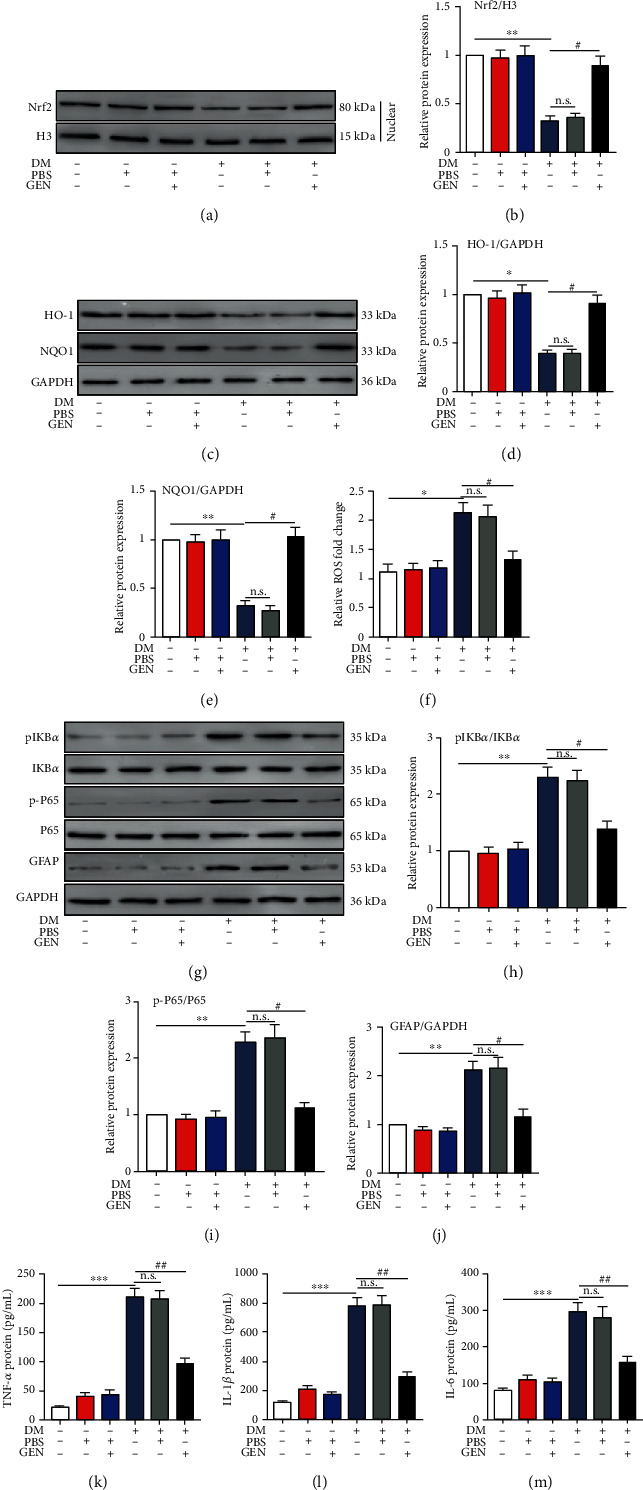
GEN-mediated activation of the Nrf2 signaling pathway reduces NF-*κ*B activation and decreases GFAP production and inflammatory cytokine secretion in the retinas of diabetic mice. DR mouse models were treated with or without GEN. (a, b) The protein expression of Nrf2 in the nucleus was measured by western blotting. ^∗∗^*P* < 0.01 vs. the normal group and ^#^*P* < 0.05 vs. the DM group. (c–e) The protein expression of HO-1 and NQO1 was measured by western blotting. ^∗^*P* < 0.05 and ^∗∗^*P* < 0.01 vs. the normal group and ^#^*P* < 0.05 vs. the DM group. (f) ROS production was measured by an ROS assay kit. (g–j). The protein expression of I*κ*B*α*, p-I*κ*B*α*, P65, p-P65, and GFAP was measured by western blotting. ^∗∗^*P* < 0.01 vs. the normal group and ^#^*P* < 0.05 vs. the DM group. (k–m) ELISA was used to measure the protein levels of TNF-*α*, IL-1*β*, and IL-6 in the retinas of diabetic mice. ^∗∗∗^*P* < 0.001 vs. the normal group and ^##^*P* < 0.01 vs. the DM group. *n* = 4/group.

To investigate whether the Nrf2 antioxidant signaling pathway and the NF-*κ*B inflammatory pathway were involved in diabetic mice in vivo, we treated the DR mouse model with GEN. As shown in Figures 6(a) and 6(b), GEN increased the nuclear translocation of Nrf2 in the retinas of diabetic mice. In addition, the Nrf2-targeted antioxidant genes HO-1 and NQO1 were decreased in the retinas of mice with diabetes, and GEN treatment increased Nrf2 antioxidant pathway activation in diabetic mice without affecting normal mice (Figures 6(c)–6(e)). Consistent with the in vitro experiments, ROS accumulation and the expression of p-I*κ*B*α* and p-P65 were upregulated in mice with DR and were reversed by GEN treatment (Figures 6(f)–6(i)). Analyses of glial reactivity and inflammatory cytokine production are shown in Figure 6(g) and Figures 6(j)–6(m). The levels of GFAP and the secretion of TNF-*α*, IL-1*β*, and IL-6 were significantly increased in the retinas of mice with diabetes compared with those of the mice in the normal group. However, GEN decreased glial reactivity (Figures 6(g) and 6(j)) and inflammatory cytokine secretion (Figures 6(k)–6(m)) in DR mice but not in normal mice.

### 3.8. GEN Decreases Hyperglycemia-Induced Damage to the BRB

**Figure 7:**
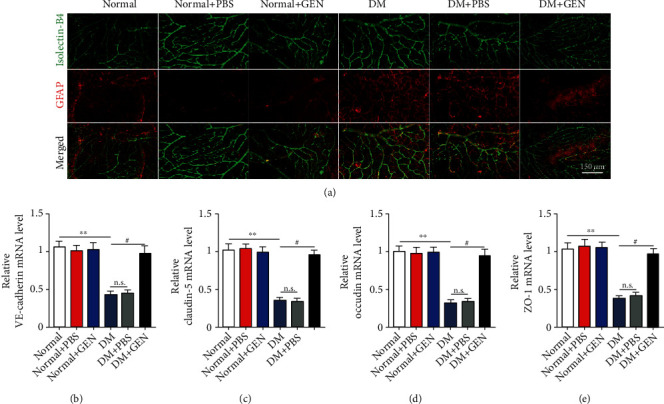
GEN decreased hyperglycemia-induced damage to the blood-retinal barrier (BRB). The mice in the normal and DM groups were treated with GEN. (a) Immunofluorescence was used to detect isolectin B4 (green) and GFAP (red) in the whole-mount retinas. White arrows show the exudate. (b–e) qRT-PCR was used to measure the mRNA expression of junction proteins, including VE-cadherin, claudin-5, occludin, and ZO-1, in the retinas of mice. *n* = 4/group.

Previous studies have shown that glial activation followed by inflammatory cytokine secretion are important factors that damage the BRB in the diabetic retinas [30]. We then investigated the effect of GEN on the BRB in diabetic mice. The results showed that the retinal vessels (marked with isolectin B4) in the diabetic group were tortuous and the exudates were increased, which were reversed by GEN. Moreover, GFAP expression was increased in the retinas of DR mice, and this effect was inhibited by GEN treatment (Figure 7(a)). In DR mice, the expressions of junction proteins such as VE-cadherin, claudin-5, occludin, and ZO-1 were decreased, while this decline was abolished by GEN treatment (Figures 7(b)–7(e)).

## 4. Discussion

Chronic inflammation and oxidative stress are considered to be the key components of DR pathogenesis, which is characterized by neuronal and vascular degeneration. Hyperglycemia leads to massive ROS production in DR [31]. ROS promotes the production and activation of NF-*κ*B, which in turn translocates to the nucleus and promotes the expression of inflammatory cytokines (such as IL-1*β* and IL-6) [32]. Müller cells are the main glial cells in the retina and play a central role in retinal metabolism. These cells are highly sensitive to metabolic changes, such as those associated with diabetes [33]. Moreover, Müller cells have been widely used in the investigation of DR pathogenesis, and so we used HG-stimulated Müller cells as an in vitro model to explore the mechanism of DR. We found that under HG conditions, ROS production and NF-*κ*B pathway activation were increased in Müller cells and were significantly attenuated by the ROS scavenger NAC. Simultaneously, the expressions of the NF-*κ*B downstream gene GFAP (a glial activation marker) and inflammatory cytokines, including TNF-*α*, IL-1*β*, and IL-6, were enhanced by HG stimulation, whereas NAC obviously reversed the effect of HG, suggesting that ROS exert proinflammatory effects on Müller cells under HG conditions. Based on these findings, we conclude that HG promotes ROS production and induces NF-*κ*B signaling pathway activation, thereby increasing glial activation and inflammatory cytokine secretion in HG-stimulated Müller cells.

It has been reported that GEN exerts potent antioxidant effects to combat various oxidative stress-related diseases, such as osteoblast diseases [34], nonalcoholic fatty liver [35], and myocardial ischemia reperfusion in diabetic rats [36]. According to previous studies, GEN prevents oxidative stress-induced damage by activating the Nrf2 antioxidant pathway [20, 37]. Under oxidative stress conditions, the transcription and synthesis of Nrf2 are increased. Moreover, oxidative stress can also facilitate the dissociation of Nrf2 from the Keap1-Nrf2 complex, allowing Nrf2 to then bind with antioxidant response elements, which in turn promotes Nrf2-mediated regulation of antioxidant genes, such as HO-1 and NQO1 [38, 39]. In the present study, we explored the biological function of GEN in DR and the potential mechanisms. We found that GEN pretreatment increased Nrf2 nuclear translocation and the expression of the downstream genes HO-1 and NQO1. In addition, ROS accumulation was dose-dependently alleviated by GEN. To further verify that GEN plays a role via the Nrf2 pathway, Nrf2 was knocked down by siRNA. The results showed that the GEN-mediated promotion of HO-1 and NQO1 expression and the inhibition of ROS production was reversed by Nrf2 knockdown. Based on these results, GEN protects Müller cells from HG-induced oxidative stress by activating the Nrf2 antioxidant signaling pathway.

Previous studies have shown that a lack of Nrf2 is associated with augmented cytokine production in experimental models of brain injury [40]. The Nrf2 activator dh404 prevented an increase in diabetes-induced inflammatory mediators, including TNF-*α*, IL-6, ICAM-1, and MCP-1, in Müller cells [41]. Moreover, studies on Nrf2^−/−^ mouse embryonic fibroblasts (MEFs) showed that IKK*β* activity was increased, I*κ*B*α* phosphorylation was enhanced, and I*κ*B*α* was subsequently degraded [42]. The NF-*κ*B inflammatory signaling pathway can be regulated by Nrf2. Wang et al. confirmed that genipin, a metabolite of GEN, can activate Nrf2 and thereby inhibit NF-*κ*B activation and inflammatory mediator production in BV2 microglial cells [43]. We revealed that GEN decreased ROS accumulation by activating Nrf2. However, whether GEN is involved in inhibiting NF-*κ*B activation and inflammatory mediator production in Müller cells remains unknown. In this study, we found that GEN inhibited the activation of NF-*κ*B and the downstream gene GFAP and inflammatory cytokines (TNF-*α*, IL-1*β*, and IL-6) in a concentration-dependent manner. Furthermore, Nrf2 knockdown reversed the anti-glial and anti-inflammatory effects of GEN on HG-stimulated Müller cells, suggesting that GEN could also inhibit the activation of NF-*κ*B and the downstream gene GFAP and inflammatory cytokines (TNF-*α*, IL-1*β*, and IL-6), which are mediated through the Nrf2 antioxidant pathway.

In recent years, GLP-1R agonists have been shown to be effective and safe treatments for diabetes and diabetic complications [44]. GLP-1R activation exerts both neuroprotective and vasculotropic effects to prevent vascular leakage in the context of DR [44]. Moreover, GLP-1R is distributed diffusely in the retina [45]. Therefore, we next explored whether GEN functioned in a GLP-1R-dependent manner, since GEN is a novel agonist of GLP-1R. We confirmed that GEN treatment was beneficial and inhibited HG-induced oxidative stress and inflammation. GEN promoted Nrf2 nuclear translocation and the expression of the downstream genes HO-1 and NQO1, while EX-9 (GLP-1R antagonist) attenuated the inhibitory effect of GEN on oxidative stress. Additionally, GEN suppressed NF-*κ*B pathway activation, GFAP expression, and inflammation-associated cytokine secretion, and these effects were reversed by EX-9. To the best of our knowledge, this is the first report to confirm that the protective effects of GEN against DR are mediated through GLP-1R.

**Figure 8:**
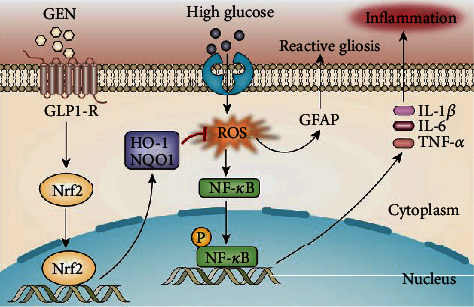
Summary of the protective effects and mechanisms of GEN in DR progression. GEN decreases HG-induced oxidative stress and inflammation, and this effect is mostly dependent on upregulating the Nrf2 antioxidant signaling pathway through GLP-1R. Nrf2 activation inhibits ROS accumulation and decreases NF-*κ*B activation and the subsequent inflammatory response.

In conclusion, we demonstrated that GEN could protect Müller cells and mice from HG-induced oxidative stress and inflammation, and the effects were mostly dependent on upregulating the Nrf2 signaling pathway through GLP-1R. The activation of Nrf2 inhibited ROS accumulation, thus decreasing NF-*κ*B activation and the subsequent gliosis and inflammatory response (Figure 8). Moreover, GEN treatment alleviated the decrease in the expression of junction proteins and may be an effective approach for the treatment of DR.

## Data Availability

The data used to support the findings of this study are available from the corresponding author upon request.
